# Nicotine has no significant cytoprotective activity against SARS-CoV-2 infection

**DOI:** 10.1371/journal.pone.0272941

**Published:** 2022-08-18

**Authors:** Fang Zheng, Elena Lian, Gaby Ramirez, Carley McAlister, Shuo Zhou, Wen Zhang, Chunming Liu, Rushika Perera, Chang-Guo Zhan

**Affiliations:** 1 Molecular Modeling and Biopharmaceutical Center, College of Pharmacy, University of Kentucky, Lexington, KY, United States of America; 2 Department of Pharmaceutical Sciences, College of Pharmacy, University of Kentucky, Lexington, KY, United States of America; 3 Center for Vector-borne Infectious Diseases, Department of Microbiology, Immunology and Pathology, Colorado State University, Fort Collins, CO, United States of America; 4 Lucille Parker Markey Cancer Center, University of Kentucky, Lexington, KY, United States of America; 5 Department of Molecular and Cellular Biochemistry, College of Medicine, University of Kentucky, Lexington, KY, United States of America; University of Catania, ITALY

## Abstract

When coronavirus disease 2019 (COVID-19) became a pandemic, one of most important questions was whether people who smoke are at more risk of COVID-19 infection. A number of clinical data have been reported in the literature so far, but controversy exists in the collection and interpretation of the data. Particularly, there is a controversial hypothesis that nicotine might be able to prevent SARS-CoV-2 infection. In the present study, motivated by the reported controversial clinical data and the controversial hypothesis, we carried out cytotoxicity assays in Vero E6 cells to examine the potential cytoprotective activity of nicotine against SARS-CoV-2 infection and demonstrated for the first time that nicotine had no significant cytoprotective activity against SARS-CoV-2 infection in these cells.

## Introduction

Coronavirus disease 2019 (COVID-19) is caused by the newly identified severe acute respiratory syndrome coronavirus 2 (SARS-CoV-2), the seventh human-specfiic coronavirus known so far [1]. In the ongoing COVID-19 pandemic, it is of interest to know whether people who smoke are at more risk of COVID-19. It has been well-known that smoking increases the risk of various serious lung diseases [2]. According to the World Health Organization (WHO), smokers are likely to be more vulnerable to COVID-19 [3]. There have been a number of clinical data reported in the literature [4–10], but controversy exists in the interpretation of the clinical data [10]. For example, in an early meta-analysis of available clinical data concerning the severity, the authors concluded that “*active smoking is not associated with severity of COVID-19*” [11]. Further reports suggested that the smoking prevalence among COVID-19 patients is lower than that among the general population [12–15], leading to a hypothesis that nicotine may be able to prevent SARA-CoV-2 infections [16, 17]. However, other researchers argued that these data should not be taken to imply that smoking or nicotine is protective for SARA-CoV-2 infection, and the observations might be the result of other unidentified factors or collider bias or even flaws in the data collection [10]. Thus, the contribution of nicotine to SARA-CoV-2 infection should be thoroughly investigated [14, 18–20]. It has also been proposed that smoking and vaping are potential risk factors for COVID-19 that are also shown to upregulate expression of angiotensin-converting enzyme 2 (ACE2), a crucial protein in COVID-19 pathogenesis [21].

Interestingly, a new clinical trial (NCT04583410) started on October 22, 2020 to examine the efficacy of nicotine in preventing COVID-19 infection in caregivers, with a hypothesis that “*nicotine may inhibit the penetration and spread of the virus and have a prophylactic effect in COVID-19 infection*” [22]. The hypothesis was based on their observation that daily active smokers are infrequent among outpatients or hospitalized patients with COVID-19 and theoretical concepts suggesting that nicotine is responsible for this protective effect *via* the nicotinic acetylcholine receptor (nAChR) [22]. The hypothesis seemed to be consistent with the concepts described in multiple reports [23–29] concerning the potential role of nicotine in COVID-19 pathology. For example, Tizabi et al. [23] suggested that “*nicotine itself*, *through its interaction with the nicotinic cholinergic system*, *as well as ACE2*, *may not only be of use in a variety of neuropsychiatric and neurodegenerative diseases*, *but may also be of potential use in COVID-19*”.

Usually, to develop a first-in-class pharmacotherapy using a given potential drug candidate corresponding to a pharmacological hypothesis, one wants to first examine whether the drug candidate has the desirable pharmacological function in cell culture. For example, Nirmatrelvir (Paxlovid) is the first FDA-approved SARS-CocV-2 3C protease (main protease) inhibitor used to treat COVID-19. In fact, Nirmatrelvir (also known as GC376) is a prodrug of GC373. Both GC373 and GC376 were reported a decade agao as the potent inhibiors of 3C or 3C-like proteases of picornaviruses, noroviruses, and coronaviruses [30]. During the COVID-19 pandemic, GC373 and GC376 were tested againt the SARS-CocV-2 3C protease. Knowing that GC373 and GC376 are indeed potent inhibitors of SARS-CocV-2 3C protease, these drug candidates were first tested in cell culture using Vero E6 cells to demonstrate their antiviral activity against SARS-CocV-2 [31] before moving forward to the further preclinical nd clinical drug development for treatment of COVID-19.

Based on the above background, in our study, motivated by the reported controversial discussion of the clinical data [9, 12–15], our goal was to examine whether nicotine has significant cytoprotective activity against SARS-CocV-2. To achieve the goal, we performed cytotoxicity tests in Vero E6 cells and demonstrated that nicotine had no significant cytoprotective activity against SARS-CoV-2 infection.

## Materials and methods

All media/reagent preparation and cell culture were performed in BSL-2. All work with the virus was performed in BSL-3.

### Virus stock

The virus strain used for this assay was SARS-CoV-2, USA WA 01/2020, CSU V3 3/17/2020. The stocks were obtained from BEI Resources and amplified to passage 3 in Vero E6 cells with a titer of 3.4 × 10^6^ PFU/mL. The stocks were stored at -80°C.

### Cell culture

The cell line utilized for the plaque reduction assay was Vero E6 cells (ATCC® CRL-1586). Vero E6 cells (known as kidney epithelial cells extracted from an African green monkey) were used in this study for a couple of reasons. First, kidney epithelial cells express nicotinic acetylcholine receptor [32]. Further, Vero E6 cells have widely been used in SARS-CoV-2 infection studies, including testing of potential drug candidates against SARS-CoV-2 [31, 33–36]. These cells were grown from a frozen aliquot of a laboratory working cell line. Passage number was limited to no more than 50 passages from the original aliquot. Cells were grown in T150 flasks in 1X DMEM (ThermoFisher cat. no. 12500062) supplemented with 2 mM L-glutamine (Hyclone cat. no. H30034.01), non-essential amino acids (Hyclone cat. no. SH30238.01), and 10% heat inactivated Fetal Bovine Serum (FBS) (Atlas Biologicals cat. no. EF-0500-A).

On the day previous to executing the assay, Vero E6 cells were removed from T150 flasks by trypsinization (0.25% Trypsin, Corning cat. no. 25-053-Cl) and measured for count and viability by hemocytometer in trypan blue. Cells were resuspended to 3.3 × 10^4^ cells/mL in 10% DMEM and seeded at 0.15 mL/well (5,000 cells/well) in two 96-well plates.

### Compound preparation

Nicotine was ordered from Sigma-Aldrich (St. Louis, MO), and was initially prepared as a 0.5 M stock in PBS. For the cytotoxicity assay, nicotine was tested at the following concentrations: 10, 5, 1, 0.5, 0.1, 0.05, 0.01, and 0.005 μM. This broad range of the nicotine concentration well covers the previously reported plasma nicotine levels in the smokers. For example, the known average plasma nicotine level after smoking cigarettes was 150.4 nM (range: 95.6 to 236.7 nM) [37]. The nicotine stock solution was diluted to the final test concentrations in 1X DMEM supplemented with 2 mM L-glutamine, non-essential amino acids, and 2% heat inactivated FBS (2% DMEM). A separate 2% DMEM (media control, virus control) was prepared for the control.

Media was aspirated from both the therapeutic and cytotoxicity plates prior to treatment with 100 μL of the various concentrations of nicotine or the vehicle control. The cytotoxicity plate was sealed with an AeraSeal to control for evaporation and even gas exchange and incubated at 37°C, 5% CO_2_ for 72 h. One-hour post-treatment, the therapeutic plate was transferred to BSL-3 for infection.

### Infection and collection

Prior to infection, the media from the therapeutic plate was transferred into a new 96-well plate. The therapeutic plate was then inoculated with 50 μL SARS-CoV-2 (MOI = 0.1) and incubated for 1 h at 37°C, 5% CO_2_ to allow the virus to adsorb to the cells. The inoculum was removed, and cells were washed once with PBS to remove residual virus. Cells were then re-treated with 90 μL of their previous media overlay (less than the original 100 μL to account for evaporation) before the plate was sealed with an AeraSeal. The plate was incubated for 72 h at 37°C, 5% CO_2_.

### Cytotoxicity assay

The cytotoxicity of the compounds was measured using the alamar blue reagent (resazurin). A working solution of 2% alamar blue was diluted 1:10 in 2% DMEM. After the media was aspirated from the cytotoxicity plate, 100 μL of the alamar blue mixture was dispensed to each well. The plate was incubated for approximately 2 h, until the reagent changed color from blue to purple.

The alamar blue reagent was transferred from the cytotoxicity plate to a new 96-well plate to avoid interference from autofluorescence by the cells. Readings were obtained by the VICTOR 1420 Multilabel Plate Reader (Perkin Elmer) with excitation at 560 nm and emission at 590 nm.

### Imaging cytometry

Cell viability and cytoprotection post-infection was measured following acridine orange (AO)/propidium iodide (PI) staining. The stock AO/PI solution was diluted 1:10 in PBS. After the supernatant was collected from the infection plate, 25 μL of the diluted stain was dispensed to each well. After a few seconds, 75 μL PBS was added to each well to increase the total volume to 100 μL and enhance staining of the center of the wells.

The plate was imaged using the Celigo Image Cytometer (Nexcelom Bioscience) and image analysis was performed using the Celigo software. The expression analysis application–Target 1+2+3 –was used to acquire images. Target 1 (brightfield), target 2 (AO–green [Ex. 483/32 nm, Em. 536/40 nm]), and target 3 (PI–red [Ex. 531/40 nm, Em. 629/53 nm]). The image-based autofocus was used to focus the cells in the brightfield channel before focus offsets were applied for each channel. Focus offsets were -4 μm, -16 μm, -24 μm for the brightfield, green (AO), and red (PI) channels, respectively.

After images were acquired for each channel, the analysis parameters for the fluorescence intensities of the green (AO) and red (PI) channels were adjusted to remove background fluorescence from the analysis. The fluorescence intensities correspond to a pixel intensity from 0–255, and the lower threshold for fluorescence intensity was adjusted to 30 and 25 for the green and red channels, respectively. The image analysis generated total cell counts, occupied area, and average intensities for each of the channels.

### Observation of cytotoxicity

After 72 h treatment, cell densities and general cell health appeared visually similar between all treatments and the 2% DMEM controls. Cell density was approximately confluent.

### Observation of infection

Following 72 h treatment, cytoprotection was not visually observed under the light microscope. Cytopathic effect (CPE) was present in all treatments and at a comparable level to that of the virus only control.

### Statistical analysis

Statistical analysis of the cytotoxicity data was performed using GraphPad Prism Software version 9.4.0 for Mac OS x (GraphPad Software, La Jolla California USA). A one-way ANOVA with Turkey’s multiple comparison test was performed for each graph with the treatments compared to the 2% DMEM control (ns = not significant, *p*>0.05).

### Additional control activity experiment–biological activity of nicotine in A549 cells

A549 lung cancer cells were cultured in DMEM/F12 supplemented with 2 mM L-glutamine and with 10% Fetal Bovine Serum (Sigma F0926) at 3°C with 5% CO_2_ atmosphere in a water jacketed incubator (NuAire, Plymouth, MN). Cells were incubated in 6-well plates and were treated with nicotine (2 or 10 mM) or the vehicle for 48 hours. Cells were lysed in 500 μl of lysis buffer: 50 mM HEPES, 100 mM NaCl, 2 mM EDTA, 1% (v/v) glycerol, 50 mM NaF, 1 mM Na_3_VO_4_, 1% (v/v) Triton X-100, with protease inhibitors (Sigma, P8340). The cell lysates were centrifuged (18,000 *g*, 10 min) and then 6X protein loading buffer was added to the supernatants. The samples were boiled for 3 min and analyzed by standard Western blotting methods [38, 39]. Phospho-p44/42 MAPK (Erk1/2) antibody was purchased from Cell Signaling Technology (#9101); GAPHD antibody was purchased from Proteintech (60004-Ig).

## Results

**Fig 1:**
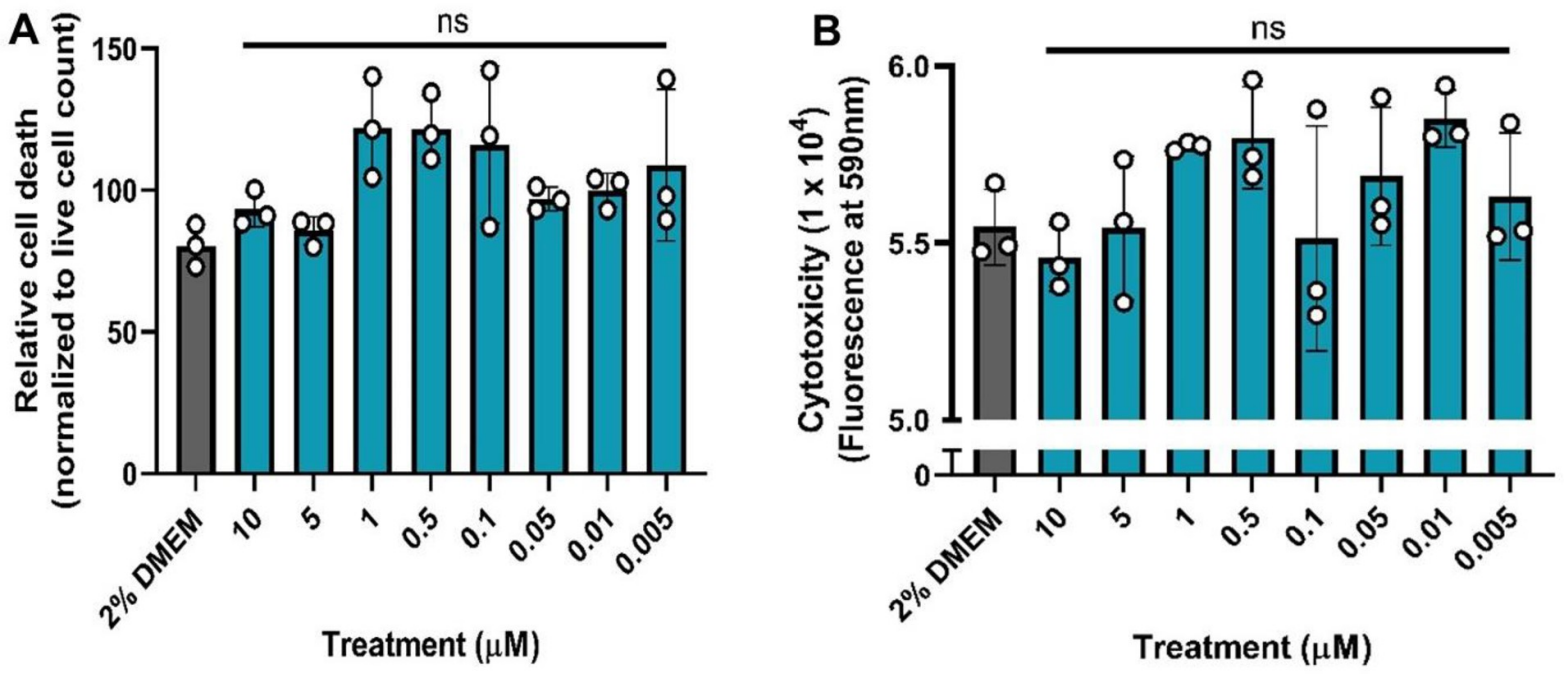
Cytotoxicity data: (A) Cytoprotection of nicotine–the efficacy of the treatment was evaluated 72 h post-infection by quantifying cell death; (B) Cytotoxicity was assessed 72 h post-treatment using alamar blue. The controls are colored in grey, and the nicotine treatments are colored in teal. A one-way ANOVA comparisons test was performed for each graph with the treatments compared to the 2% DMEM control (ns = not significant).

Motivated by reported meta-analyses [12–15], we decided to perform cytotoxicity tests on nicotine against SARS-CoV-2 infection in Vero E6 cells (known as kidney epithelial cells extracted from an African green monkey) as the Vero E6 cells have widely been used in SARS-CoV-2 infection studies in testing potential drug candidates against SARS-CoV-2 [31, 33–36], including GC373 and GC376, the first FDA-approved SARS-CoV-2 3C protease inhibitor for treatment of COVID-19. Depicted in **Fig 1** (Graph Pad Prism 8.4.1 software was used) are the cytotoxicity data, including the activity of nicotine against SARS-CoV-2 infection (**Fig 1A**) and cytotoxicity of nicotine (**Fig 1B**). Cytoprotection was calculated as cell death relative to the live cell count. Cell death was measured by the number of cells stained with PI, and live cells were measured *via* AO staining.

The following equation was used to calculate relative cell death:

(deadcellcount[PI]/livecellcount[AO])×100=Percentdead (1)

As seen in **Fig 1A**, for nicotine, none of the concentrations conferred cytoprotection against SARS-CoV-2 infection in Vero E6 cells. Meanwhile, compared to the media control (2% DMEM), the nicotine treatment was not cytotoxic (**Fig 1B**).

**Fig 2:**
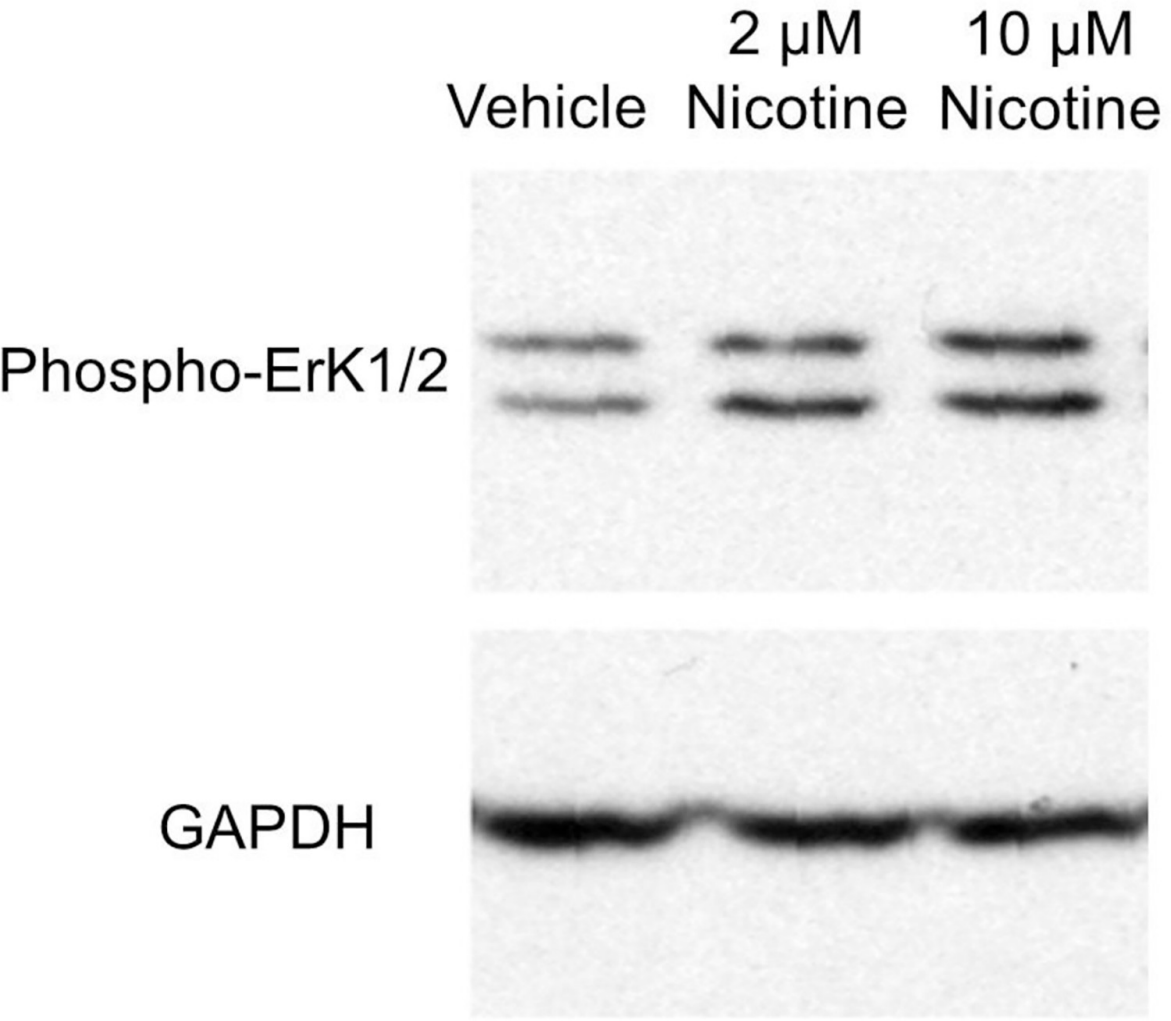
Nicotine activated Erk1/2 in A549 cells. A549 cells were treated with nicotine or vehicle for 48 hours, showing that nicotine induced Erk1/2 phosphorylation. GAPDH was analyzed as a loading control.

To test whether nicotine was active in the cell growth media and cell culture condition using the DMEM supplemented with 2 mM L-glutamine, we treated A549 lung cancer cells with the nicotine (2 or 10 μM) or the vehicle control (0 μM nicotine) for 48 hours. It has been reported that nicotine can activate the Erk in multiple cell lines *via* promotion of the Erk phosphorylation [40–45]. We found that nicotine indeed induced Erk1/2 phosphorylation in A549 cells as shown in **Fig 2**, suggesting that nicotine was active in the cell culture media DMEM supplemented with 2 mM L-glutamine.

## Discussion

According to reported clinical data and meta-analyses [12–15], within the COVID-19 patients, the smoker group has a significantly higher ratio of severe COVID-19 compared to the non-smoker group. Notably, the significantly higher ratio of severe disease in the smoker group is mainly attributed to the past smokers. Smokers, particularly the past smokers, are at significantly higher risk of severe COVID-19 disease. On the other hand, the reported meta-analyses [12–15] also revealed the significantly decreased COVID-19 occurrence in the active smoker group, which seemed to suggest some potential, but questionable [10], beneficial effects of active smoking. This suggestion motivated us to test the potential cytoprotective activity of nicotine against SARS-CoV-2 infection in Vero E6 cells, the same cell line used to determine antiviral activity of GC373 and GC376 (known as Nirmatrelvir or Paxlovid now) against SARS-CoV-2 [31]. The highest concentration of nicotine used in the cytoprotective activity tests was 10 μM (or 10,000 nM), which is much higher than the previously known average plasma nicotine level of 150.4 nM (range: 95.6 to 236.7 nM after smoking cigarettes [37]. We understand that the plasma concentration of nicotine might not represent the actual nicotine concentration for the cell exposure, depending on its biodistribution. Nevertheless, as well known, nicotine is absorbed and distributed quickly in the body [46]. According to nicotine biodistribution and pharmacokinetics data reported in literature [47, 48], the nicotine concentrations observed in other tissues were not dramatically higher than the corresponding nicotine concentrations in the plasma. In other words, it is reasonable to assume that the nicotine concentrations for the cell exposure will likely not higher than 10 μM in the normal situations of nicotine intake or smoking, although it would be interesting to study this issue further in the future. According to the cytotoxicity data obtained, even at a concentration as high as 10 μM, nicotine has no significant cytoprotective activity against SARS-CoV-2 infection, whilst nicotine at a concentration of 2 or 10 μM in the DMEM media supplemented with 2 mM L-glutamine has its normal function in promotion of the Erk phosphorylation.

Our cytotoxicity data are consistent with a most recently published review/comment [10] pointing out some potential flaws or bias of some reported studies in this topic. Partciularly, according to the review/comment [10], there were also a variety of methodological issues in the clinical data collection. In other words, the collected clinical data used to perform meta-analyses are questionable.

In addition, even if the collected clinical data can be trusted, our cytotoxicity data implies at least two other possibilities. First, reported meta-analyses of the COVID-19 occurrence were based on the overall tobacco smoking prevalence for a given country (China or USA), without accounting for detailed demographic factors such as age, body mass index, and living condition/socioeconomic status, because these more detailed demographic data are not available. For example, people living below the poverty level and people having lower levels of educational attainment have higher rates of cigarette smoking than the general population in the US [49]. Without accounting for these possibly important confounders, the results of a meta-analysis could be misleading. For the other possibility, even if active smoking really can decrease the occurrence of COVID-19 disease as shown in the analysis, the ingredient(s) responsible for the beneficial effects must be compound(s) other than nicotine absorbed *via* smoking. Hence, the experimental results obtained in this study do not appear to support the ongoing Phase 3 clinical trial (NCT04583410) using the nicotine patch for its prophylactic effect in COVID-19 infection.

Finally, we would like to point out that our current study also has its limitations because it is limited to the use of only one cell line–Vero E6 cells, the same cell line used to determine antiviral activity of GC373 and GC376 (known as Nirmatrelvir or Paxlovid now) against SARS-CoV-2 [31]–without testing nicotine *in vivo*. It might also be interesting to test nicotine further in other cell lines and/or *in vivo* studies against SARS-CoV-2 infection in the future.

## Supporting information

S1 File (PDF)
