# Supplementary material for: Nicotine has no significant cytoprotective activity against SARS-CoV-2 infection
Source: PLoS One. 2022 Aug 18;17(8):e0272941. doi: 10.1371/journal.pone.0272941 (PMC9387791; doi:10.1371/journal.pone.0272941)

Supporting Information

Nicotine has no significant cytoprotective activity against SARS-CoV-2 infection

Fang Zheng,[1,2] Elena Lian,[3] Gaby Ramirez,[3] Carley McAlister,[3] Shuo Zhou,[2] Wen Zhang,[4,5] Chunming Liu,[4,5] Rushika Perera,[3] and Chang-Guo Zhan[1,2]

[1]*Molecular Modeling and Biopharmaceutical Center, College of Pharmacy, University of Kentucky, 789 South Limestone Street, Lexington, KY 40536.*

[2]*Department of Pharmaceutical Sciences, College of Pharmacy, University of Kentucky, 789 South Limestone Street, Lexington, KY 40536.*

[3]*Center for Vector-borne Infectious Diseases, Department of Microbiology, Immunology and Pathology, Colorado State University, Fort Collins, CO 80523.*

[4]*Lucille Parker Markey Cancer Center, University of Kentucky, Lexington, KY 40536.*

[5]*Department of Molecular and Cellular Biochemistry, College of Medicine, University of Kentucky, Lexington, KY 40536.*

Running Title: Nicotine is not cytoprotective against SARS-CoV-2

Correspondence:
Chang-Guo Zhan, Ph.D.
Director, Molecular Modeling and Biopharmaceutical Center (MMBC)
Director, Chemoinformatics and Drug Design Core of CPRI
University Research Professor
Endowed College of Pharmacy Professor in Pharmaceutical Sciences
Professor, Department of Pharmaceutical Sciences
College of Pharmacy
University of Kentucky
789 South Limestone Street
Lexington, KY 40536
Phone: 859-323-3943
FAX: 859-257-7585
E-mail: zhan@uky.edu

Nicotine (μM) cytoprotection activity data for Figure 1A

2% DMEM	10μM	5μM	1μM	0.5μM	0.1μM	0.05μM	0.01μM	0.005μM
87.93607	88.43975	88.39885	140.2076	111.0186	87.06897	101.3409	104.0137	139.3057
80.44314	91.04242	80.09447	121.4879	134.4987	119.2192	93.15804	93.1105	89.51033
73.11986	100.2888	88.82863	104.5408	119.7156	142.307	96.43155	102.9923	97.90193

Nicotine (μM) cytotoxicity data for Figure 1B

2% DMEM	10μM	5μM	1μM	0.5μM	0.1μM	0.05μM	0.01μM	0.005μM
54753	54352	53312	57610	57441	52971	56031	58001	55197
56690	53779	57353	57841	59611	58777	59117	59448	58396
54923	55596	55595	57747	56886	53654	55519	58087	55356

Uncropped gel for Phospho-Erk1/2 in Figure 2

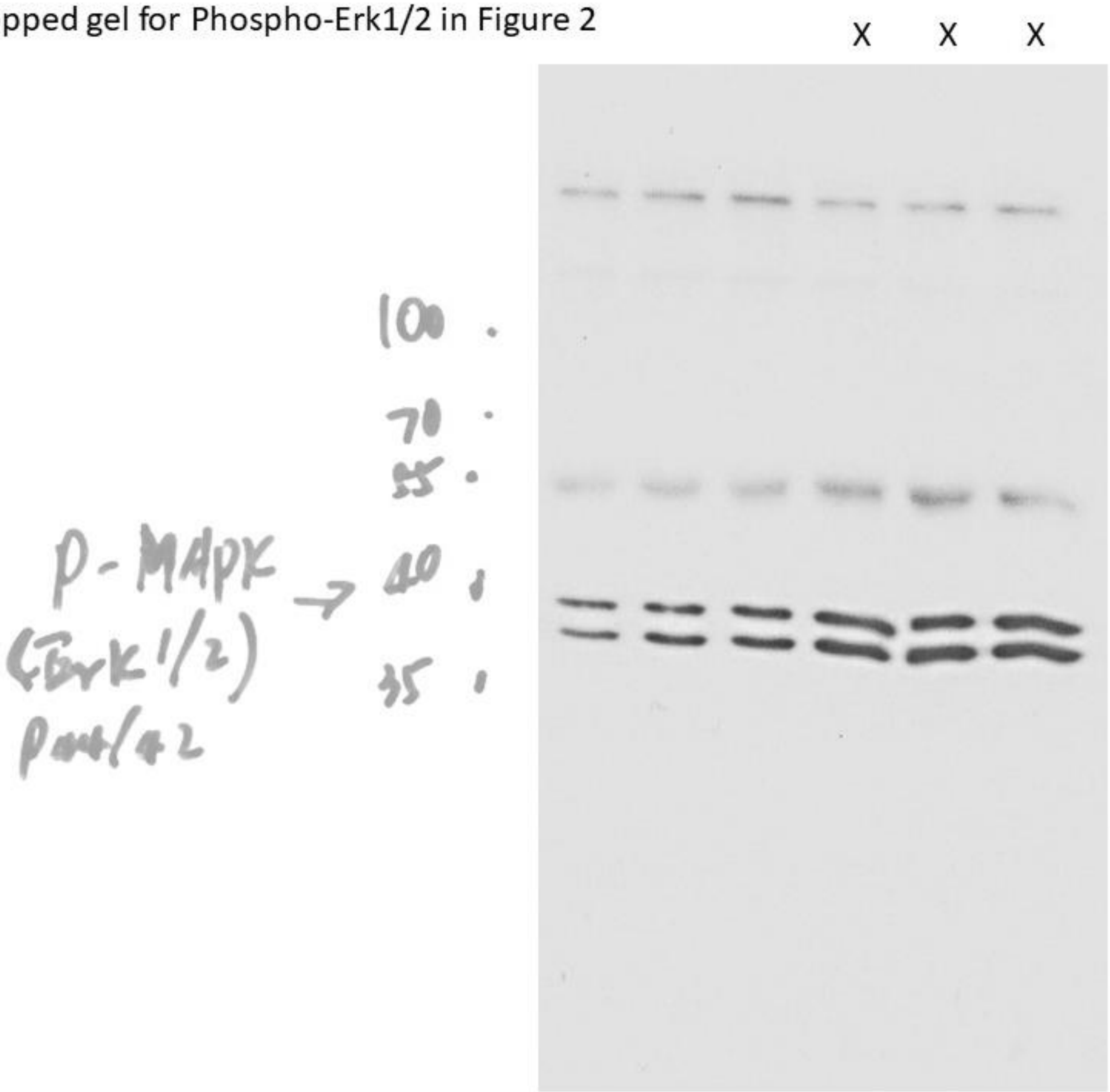

Uncropped gel for GAPDH in Figure 2

X X X X X X

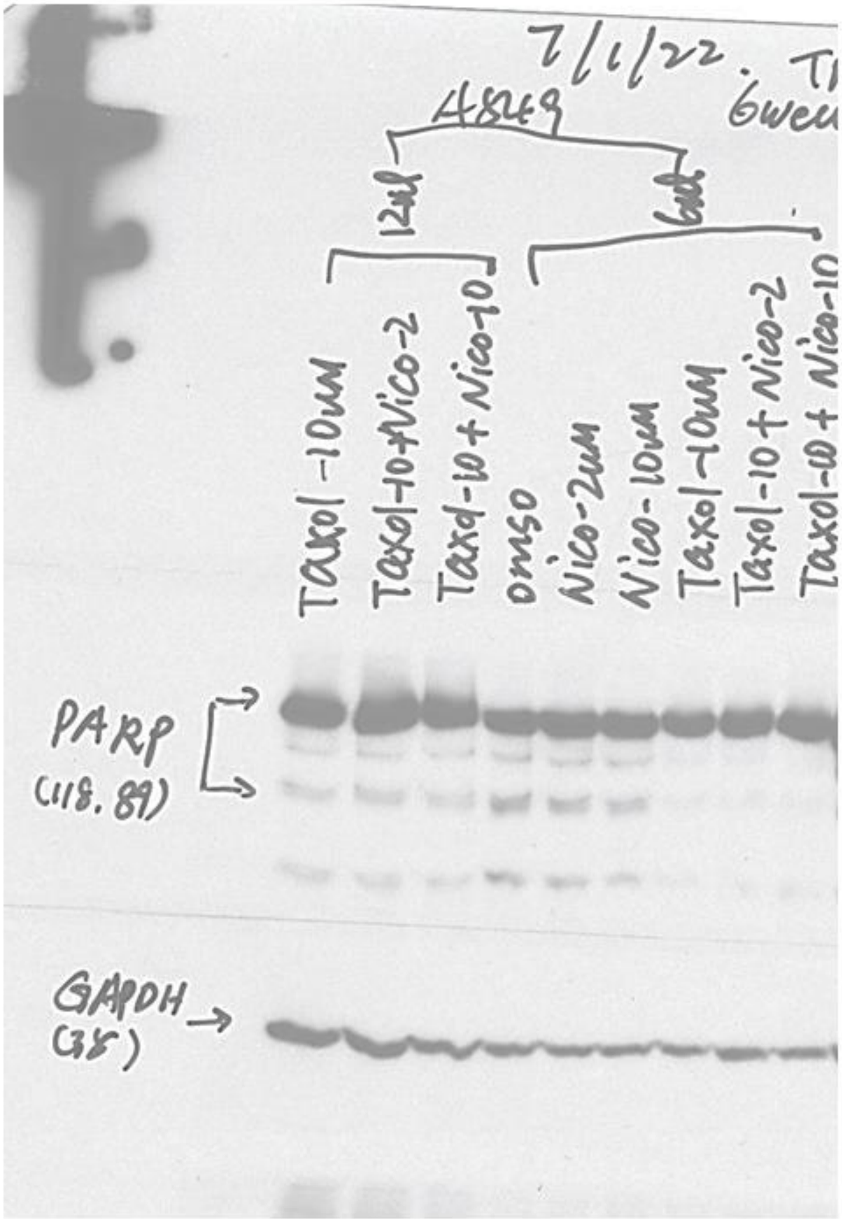

Supplement: S1 File — (PDF) [file pone.0272941.s001.pdf]
